# Hot-Melt Extrusion-Based Dexamethasone–PLGA Implants: Physicochemical, Physicomechanical, and Surface Morphological Properties and In Vitro Release Corrected for Drug Degradation

**DOI:** 10.3390/pharmaceutics16070895

**Published:** 2024-07-04

**Authors:** Alireza (Allen) Ghaffari, Brock A. Matter, Rachel R. Hartman, David W. A. Bourne, Yan Wang, Stephanie Choi, Uday B. Kompella

**Affiliations:** 1 Department of Pharmaceutical Sciences, University of Colorado Anschutz Medical Campus, Aurora, CO 80045, USA; a.ghafari51@gmail.com (A.G.); brock.matter@cuanschutz.edu (B.A.M.); rachel.r.hartman@cuanschutz.edu (R.R.H.); david.bourne@cuanschutz.edu (D.W.A.B.); 2 NextHerbal Labs, LLC, Aurora, CO 80045, USA; 3 Center for Drug Evaluation and Research, Food and Drug Administration, White Oak Campus, Silver Spring, MD 20993, USA; yan.wang3@fda.hhs.gov (Y.W.); stephaniechoi1122@gmail.com (S.C.); 4 Department of Ophthalmology, University of Colorado Anschutz Medical Campus, Aurora, CO 80045, USA; 5 Department of Bioengineering, University of Colorado Anschutz Medical Campus, Aurora, CO 80045, USA; 6 Colorado Center for Nanomedicine and Nano Safety, University of Colorado Anschutz Medical Campus, Aurora, CO 80045, USA

**Keywords:** biodegradable, corticosteroid, dexamethasone, hot-melt extrusion (HME), LC-MS/MS, intravitreal, ocular drug delivery systems, poly-(lactide-co-glycolide) PLGA, implants, sustained drug release

## Abstract

Developing bioequivalent (BE) generic products of complex dosage forms like intravitreal implants (IVIs) of corticosteroids such as dexamethasone prepared using hot-melt extrusion (HME), based on biodegradable poly (lactide-co-glycolide) (PLGA) polymers, can be challenging. A better understanding of the relationship between the physicochemical and physicomechanical properties of IVIs and their effect on drug release and ocular bioavailability is crucial to develop novel BE approaches. It is possible that the key physicochemical and physicomechanical properties of IVIs such as drug properties, implant surface roughness, mechanical strength and toughness, and implant erosion could vary for different compositions, resulting in changes in drug release. Therefore, this study investigated the hypothesis that biodegradable ophthalmic dexamethasone-loaded implants with 20% drug and 80% PLGA polymer(s) prepared using single-pass hot-melt extrusion (HME) differ in physicochemical and/or physicomechanical properties and drug release depending on their PLGA polymer composition. Acid end-capped PLGA was mixed with an ester end-capped PLGA to make three formulations: HME-1, HME-2, and HME-3, containing 100%, 80%, and 60% *w*/*w* of the acid end-capped PLGA. Further, this study compared the drug release between independent batches of each composition. In vitro release tests (IVRTs) indicated that HME-1 implants can be readily distinguished by their release profiles from HME-2 and HME-3, with the release being similar for HME-2 and HME-3. In the early stages, drug release generally correlated well with polymer composition and implant properties, with the release increasing with PLGA acid content (for day-1 release, R^2^ = 0.80) and/or elevated surface roughness (for day-1 and day-14 release, R^2^ ≥ 0.82). Further, implant mechanical strength and toughness correlated inversely with PLGA acid content and day-1 drug release. Drug release from independent batches was similar for each composition. The findings of this project could be helpful for developing generic PLGA polymer-based ocular implant products.

## 1. Introduction

As stated in FDA (Food and Drug Administration) statutes and regulations, generic drug products generally must show pharmaceutical equivalence and bioequivalence (BE), among other things, to the reference listed drug (RLD) to obtain approval [1,2,3]. For generic ophthalmic topical solutions that are qualitatively (Q1) and quantitatively (Q2) the same as the RLDs, the BE is considered self-evident. For complex ophthalmic products (e.g., implants), BE cannot be established solely based on the Q1/Q2 sameness of a proposed test formulation. Ophthalmic implants exhibit a complex drug release behavior involving drug diffusion as well as polymer degradation. These implants, even when they are considered Q1/Q2 to their RLDs, can have varying physicochemical and physicomechanical properties due to differences introduced during manufacturing, which in turn may affect ocular drug release and bioavailability. Accordingly, it is crucial to understand the relationships between the physicochemical, physicomechanical, and surface morphological properties and in vitro drug release of implants and their effect on ocular bioavailability. Further, there is no clear understanding of the influence of formulation and manufacturing parameters on drug release from dexamethasone implants. It is possible that properties such as implant surface area, porosity, tensile strength, and polymer degradation can differ for Q1/Q2 implants, resulting in differences in drug release and bioavailability.

The overall goal of the studies herein was to test the hypothesis that biodegradable dexamethasone-loaded ophthalmic implants with slightly different compositions could differ in physicochemical, physicomechanical, and morphological properties and drug release. To this end, we used a single 50:50 poly (lactide-co-glycolide) (PLGA) acid end-capped polymer or the combination of the acid end-capped polymer and a 50:50 poly (lactide-co-glycolide) (PLGA) ester end-capped polymer. Further, both polymers were supplied by the same manufacturer, had the same inherent viscosity (dL/g), and used the same implant manufacturing process to prepare all the formulations and batches of implants for the studies herein. This manufacturing process was a scalable single-pass hot-melt extrusion (HME) process.

## 2. Materials and Methods

### 2.1. Materials for Implant Manufacture

Implants were composed of micronized dexamethasone (Dex, D4902, from Shanxi Jinjin Chemical Co., Ltd., Hejin, China; about 2 µm in size) which was mixed with LACTEL^®^ absorbable PLGA polymers, a 50:50 PLGA acid end-capped polymer (B6013-1), and a 50:50 PLGA ester end-capped polymer (B6017-1), purchased from Durect Corporation, Birmingham, AL, USA, which is now part of Evonik Industries. These polymers were similar in nature to those used in the FDA-approved Ozurdex product. Both PLGA polymers have a similar lactic–glycolic ratio (L:G monomer ratio) and inherent viscosities (IVs) of 0.15 to 0.25 dL/g, but the polymers exhibit different degradation properties because of the polymer’s end-cap (e.g., the acid end-capped polymer degrades faster because of its hydrophilic nature as opposed to the hydrophobic nature of the ester end-capped polymer) [4,5,6]. By using these two polymers, it was theorized that formulations could be manufactured with fast, intermediate, and slow in vitro release characteristics.

### 2.2. Material for LC-MS/MS Analysis

2-propanol, acetonitrile, ammonium formate, formic acid, and sodium dodecyl sulfate (SDS) were purchased from Thermo Fisher Scientific, Waltman, MA, USA. Dexamethasone-4, 6, 21, 21-d was purchased from CDN Isotopes, Quebec, Canada. Any other chemicals used in the studies herein were analytical grade and obtained from commercial lab suppliers.

## 3. Methods—Implant Manufacture

### 3.1. Implant Formulations

Implants with theoretically fast, intermediate, and slow drug release characteristics were manufactured in 10 g batch size formulations with/without dexamethasone using a single-pass hot-melt extrusion (HME) technique, Table 1. The composition of the active pharmaceutical ingredient (API), micronized dexamethasone (Dex), was fixed in each of the drug-loaded batches at 20% (*w*/*w*). Depending on the desired release characteristics, either one or a mixture of the PLGA polymers was incorporated into the batch. Four batches were prepared for each of the six formulations.

### 3.2. Implant Manufacture by Hot-Melt Extrusion (HME)

Implant batches with/without dexamethasone were prepared with a lab-scale twin-screw HAAKE MiniCTW Hot-melt Extruder (Thermo Fisher Scientific, Karlsruhe, Germany) using a single-pass process described below. The micronized dexamethasone–PLGA polymer batch mixtures were prepared by weighing out the materials in the selected proportions from Table 1 and then grinding each mixture into a fine powder. These mixtures were slowly transferred individually into the preheated extruder barrel process chamber, (100 °C), screw barrel-mixed for 10 min, and then extruded at a constant torque value of 0.2 Nm for 10 min through a 0.3 mm orifice die plate. The extrudate was guided onto the conveyor belt of the M22 Transport Conveyor machine (Profilex Systems, S.A., Troisvierges, Luxembourg) to form 6- to 8-inch-long strands with diameters that ranged from 0.375 to 0.420 mm. Strands with diameters of 0.4 mm were selected to be cut into 6 mm implant lengths for testing. The hot-melt extruder process was optimized to produce more consistent implant diameters as discussed elsewhere [7]. The 0.4 mm diameter specification was selected because this diameter fits perfectly inside the ultra-thin-walled 22-gauge needles used for IVT injections into the eye [8]. When necessary, the dimensions and masses of implants were obtained using a Digital Micrometer (Mitutoyo Corporation, Kawasaki, Kanagawa, Japan) and an analytical balance (Denver Instruments, Denver, CO, USA).

### 3.3. Methods—Physicochemical Characterization

#### 3.3.1. Implant Dexamethasone Content Determined by HPLC

To determine the actual dexamethasone content, 10 implants from HME-1, HME-2, and HME-3 weighing around 1 mg were placed separately into vials containing 40% aqueous acetonitrile, sonicated for 1–3 min, and then diluted 10x with the same solution. The drug concentration in each sample was determined using a Waters^TM^ HPLC (High-Performance Liquid Chromatography) System (Water Corporation, Milford, MA, USA) with a 600 Controller, 717 plus Autosampler, and 996 Photodiode Array Detector. Samples were injected (50 µL) onto an Eclipse Plus C18, 4.6 × 150 mm, 5 µm column (Agilent, Santa Clara, CA, USA). The column was kept at room temperature (~22 °C) and eluted isocratically at 1 mL/min with 40% aqueous acetonitrile. Data were collected at 242 nm. A standard curve from 5 to 30 was generated, and the percent dexamethasone content was then calculated.

#### 3.3.2. Thermal Analysis—Using Differential Scanning Calorimetry (DSC)

For differential scanning calorimetry, implants HME-1, HME-2, and HME-3 along with their formulation components were analyzed to determine thermal patterns as a function of temperature [9,10,11]. Thermal data were collected using a Q2000 Differential Scanning Calorimeter (DSC) (TA Instruments, New Castle, DE, USA). The analysis was conducted in a nitrogen environment with a purge rate of 50 mL/min. Dexamethasone implants and PLGA polymers were pulverized; 1 to 5 mg of each sample was sealed in aluminum weighing pans. Instrument calibration was performed before running the samples using sapphire and indium standards. Samples were processed using a seven-step DSC method. The upper temperature limits were set at 200 °C for PLGA polymers and 268.5 °C for the micronized dexamethasone because higher temperatures in either case would have caused the formulation components or drug to degrade. After processing, the transition temperatures were analyzed using the instrument’s Universal Analysis software (version 2000).

### 3.4. Methods—Physicomechanical Characterization

#### 3.4.1. Melt Viscosity

Melt viscosity studies were performed on formulations with/without dexamethasone in 10 g batch sizes. Each formulation was processed individually through the extruder. Test parameters were set into the extruder software, which included a fixed screw speed of 20 rpm, mixing at three different temperatures (60 °C, 80 °C, and 100 °C) for at least 3 min, while the software recorded 120 mean torque values. These values were an indication of each formulation’s melted viscosity.

#### 3.4.2. Influence of Humidity

##### Three-Point Bend (TPB) Tests Using a Texture Analyzer

Three-point bend (TPB) tests, also known as three-point flexural tests, were used to compare the mechanical properties of the strength and toughness of implant formulations stored under low- or high-relative humidity conditions, (LRH or HRH), respectively [12,13,14,15,16,17]. To conduct the TPB tests, strands from each formulation were cut into six 3 cm pieces and placed in centrifuge tubes stored inside two custom-made humidifiers. These humidifiers consisted of two desiccators set up to simulate the different storage conditions; the 1st contained a saturated sodium chloride solution so that an HRH > 75% was created, and the 2nd contained Drierite^®^ regular (CaSO4) desiccant (Alfa Aesar, Haverhill, MA, USA) so that an LRH < 30% was created. Each desiccator was stored at room temperature while connected to a vacuum line. Using a Libero THi1-Y V3.24 Data logger (Elpro-Buchs AG, Buchs, Switzerland), the temperature and relative humidity inside the humidifiers were monitored for a 96 h period. After 96 hrs, the strands were removed to conduct TPB analysis using a Texture Analyzer (Model: TA-XT plus, Stable Micro Systems, Surrey, UK); test parameters are listed in Table 2.

##### Moisture Adsorption of Implants under High Relative Humidity

Along with performing TPB tests on implants stored under high-relative humidity conditions, the moisture adsorption of implant compositions with/without dexamethasone was also determined. Representative samples were removed after 24, 48, 72, and 96 h from the humidifier and weighed to determine moisture adsorption.

### 3.5. Methods—Surface Morphological Properties

#### 3.5.1. Atomic Force Microscopy (AFM)

For atomic force microscopy (AFM), implants from the dexamethasone-loaded formulations were firmly mounted on a glass disc. Additionally, to observe the surface morphology of implants during dexamethasone release studies, implants from these batches were placed in 250 mL bottle containing 100 mL of PBS and agitated on a MaxQ 4000 shaker incubator (Thermo Fisher Scientific, Waltham, MA, USA) with a rotational speed of 150 rpm at 37 °C. On day-1 and day-14 after exposure to the media, implants were removed, placed into a vacuum desiccator to dry for 24 hrs, and then mounted on a glass disc for AFM. The AFM images were captured using a Multi-mode Atomic Force Microscope (Bruker Corporation, Santa Barbara, CA, USA) and processed using NanoScope analysis software (version 1.50). All images were captured in air using the Peak Force Tapping (PFT)^®^ mode to reduce sample–probe interaction forces [18,19,20], Scan Analyst-Air-HR Bruker AFM probes with spring constants ranging from 0.5 to 0.8 N/m, and the peak force set points that were automatically adjusted and varied from 700 pN to 1.7 nN for different samples.

#### 3.5.2. Polarized Light Microscopy (PLM) on Implant Films

To prepare specimens for PLM analysis, transparent sample films were created using a melt-congealing technique [21,22,23]. A custom-made glass coverslip cell was produced by melting implants individually, (HME-1, HME-2, and HME-3 with dexamethasone and HME-4, HME-5, and HME-6 without dexamethasone) at 110 °C while positioned on a Kofler Hot Bench, Type WME (Reichert-Jung Heizbank, München, Germany), and then compressing the molten material. Using an Eclipse E400 microscope (Nikon Polarized Light Microscope, Tokyo, Japan), the film casts were examined under 10× magnification. Images were then obtained using a Camedia C-5050 Zoom digital camera (Olympus, Melville, New York, NY, USA) attached to the microscope eyepiece.

#### 3.5.3. Scanning Electron Microscopy (SEM)

The implants used for SEM imaging were fresh HME-1, HME-2, and HME-3 formulations, as well as representative implant samples from these batches after exposure to the PBS release media for day-1 to day-14. All the samples were fixed with an adhesive sheet to aluminum stubs (Ted Pella, Redding, CA, USA) and platinum-coated using an Auto Sputter Cressington 108 Coater (Ted Pella, Redding, CA, USA) with a 30 s argon gas flow. The structural surface morphology of the samples was then examined by using SU 3500 SEM (Hitachi High Technologies America, Schaumburg, IL, USA) at 10 kV and by examining the samples at 150×, 230×, and 500× magnifications.

#### 3.5.4. Powder X-ray Diffraction (XRD) Analysis

The XRD analysis was performed using a D2 Phaser desktop diffractometer (Bruker AXS, Inc., Madison, WI, USA). Before the analysis, each sample was pulverized until a fine powder was obtained. Analysis was performed using a Ni-filtered Cu Kα monochromatic X-ray radiation at 30 kV with an intensity of 10 mA. Samples were scanned at a 0.02° angle, at a rate of 0.25 s per step for 20 steps over a temperature range from 4 to 40 °C. The presence or absence of visible peaks was considered an indication of crystalline or amorphous forms of the sample [24,25,26,27,28,29,30,31].

### 3.6. Methods—In Vitro Release Testing

#### 3.6.1. Large-Volume (100 mL) In Vitro Release Studies

In vitro release studies were performed in quadruplicate using samples from the three batches with different formulations (HME-1, HME-2, and HME-3) to determine dexamethasone release and degradation profiles. Each dexamethasone-loaded implant was placed into an amber 250 mL Pyrex bottle containing 100 mL of phosphate buffer solution, (PBS pH 7.4), then placed in holders on a MaxQ 4000 Shaker Incubator (Thermo Fisher Scientific, Waltham, MA, USA) set with a rotational speed of 150 rpm and temperature at 37 °C under dark study conditions (i.e., to ensure complete dark conditions, the incubator lid was covered with aluminum foil). A 1 mL sample aliquot (1% total volume) was removed from each bottle at the following time points: 1, 7, 14, 21, 28, 35, 42, 49, 56, 63, and 71 days, placed in pre-labeled sample vials. Fresh PBS was added to replace the 1 mL sample volume that was removed. Collected samples were stored at −80 °C until samples from all the time points were collected. Then, 75 µL aliquots were removed from each sample, spiked with d4-dexamethasone (5 µL), and transferred to well plates. The analysis was performed on a Prominence HPLC (Shimadzu, Kyoto, Japan) coupled to a QTrap 4500 mass spectrometer (AB Sciex, Farmingham, MA, USA) [32].

#### 3.6.2. HPLC-ESI+-MS/MS Detection and Quantification of Dexamethasone Release

Several researchers have described analytical methods for dexamethasone stability determination. Our lab developed a stable isotope dilution LC-MS/MS method that could detect/quantify dexamethasone and 13 of its degradation products in a single run described in length elsewhere [32,33,34,35,36]. This previously described method was used for LC-MS/MS quantitation in this work.

In brief, the mass spectrometer was operated in ESI+ mode, optimizing settings by manually tuning using an infused drug solution. The resulting optimized parameters are as follows: curtain gas, 50 psi; collision gas, high; ion spray voltage, 5500 V; source temperature, 600 °C; ion source gas 1 and 2, 30 psi; declustering potential, 40 V; and collision energy, 14 V. As for sample quantitation, it was performed in the multiple reaction mode (MRM) with dexamethasone-d4 used as an internal standard for the drug and degradation products. The analytical separation used a Phenomenex phenyl-hexyl column (3 × 100 mm, 3.5 μm) to achieve separation. This column was held at 40 °C and eluted at 0.5 mL/min with a gradient of 10% acetonitrile in 20 mM ammonium formate, pH 3.8 (A), and 10% isopropanol in acetonitrile (B). Additionally, chromatographic separation was achieved with a linear gradient (time, % of solvent B) of 0–9 min, 10.5–13.7% B; 9–17 min, 13.7–21% B; 17–22 min, 21–35% B; 22–23 min, 35–10.5% B and then isocratic for 10 min at 10.5% B to re-equilibrate the column. The concentration of dexamethasone in each sample was calculated from the ratio of the HPLC-ESI -MS/MS peak area for the drug to the peak area for dexamethasone-d4. This ratio was multiplied by the known concentration of the internal standard, then adjusted for instrument response with a calibration curve constructed with known ratios of the drug and internal standard. Because of the structural similarities, this allowed for the use of the same calibration curve for the quantitation of dexamethasone degradation products. The lower limit of quantitation for dexamethasone was 10 fmols of dexamethasone on the column with an inter-day accuracy of 103.6% ± 5.7% with a % RSD of 5.5%. The lower limit of detection was defined as a peak with a signal-to-noise ratio ≥ 5.

#### 3.6.3. Degradation Rate Constant and Concentration Correction in Phosphate-Buffered Saline Solution (PBS pH 7.4)

The dexamethasone degradation rate constant was calculated from dexamethasone standards incubated in PBS solution (pH 7.4) at 37 °C under dark study conditions. The degradation rate constant was calculated by plotting dexamethasone concentrations vs. sampling time on a semi-log scale as previously reported by our lab [32]. Additionally, when the incubations were sampled, the PBS solution removed was replaced with fresh PBS, 1% of the total volume. This meant that the dexamethasone concentration in the remaining incubation solution was reduced by 1% at each sampling time point, and if not removed, the solution would have continued to degrade. We used Equation (1) to correct for dexamethasone lost to sampling and to simulate the degradation of the removed dexamethasone sample. We started with the uncorrected degradation rate constant and then iteratively applied Equation (1) to refine the degradation rate constant [32],

(1) $${Dex}_{\left(n;\;c\right)}={Dex}_{\left(n;\;o\right)}+\left\{\left[\left({Dex}_{\left(n-1;\;o\right)}\times 1\%\right)+\left({Dex}_{\left(n-1;\;c\right)}-{Dex}_{\left(n-1;\;o\right)}\right)\right]\cdot e^{k_{d}\cdot \Delta t}\right\}$$

where “Dex” is the concentration of dexamethasone, subscript “n” is the current sample, subscript “n − 1” is the previous sample, and “Δt” is the time between sampling (t_(n)_ − t _(n−1)_).

## 4. Results and Discussion

The drug release from PLGA implants is via diffusion through the polymer- or water-filled pores that may form or change during release, degradation and erosion of the polymer, or a combination. While the PLGA polymers allow for controlled release from weeks to months with hydrophilic polymers releasing the drug more rapidly in general, the actual pattern of release is complex to predict or mimic in vitro, let alone in vivo, and depends on drug, polymer, and manufacturing properties, explaining the difficulty in developing generic products for complex drug products like implants.

Using an LC-MS/MS analytical method [32], this study investigated the influence of polymer compositions on drug release for dexamethasone–PLGA implants manufactured in batches using a single-pass hot-melt extrusion process. Micronized dexamethasone–PLGA implants were manufactured to achieve three theoretically different release profiles: fast, intermediate, and slow. The extrusion process was rapid, with the only observed process issue being a decline in the implant diameter toward the end of the process which was addressed in a study described elsewhere [7]. One of our lab’s prior studies tested dexamethasone release in a small volume (5 mL) and determined the dexamethasone concentration using UV spectrophotometry, whereas the current study used a large volume (100 mL) and an LC-MS/MS method capable of analyzing for dexamethasone and/or degradation products of dexamethasone [32]. Additionally, this study evaluated the physicochemical, physicomechanical, and surface morphological properties of dexamethasone–PLGA implants.

### 4.1. Dexamethasone Implant Manufacturing

Drug encapsulation in PLGA polymer formulations usually exhibits drug release through a combination of mechanisms, matrix diffusion through water uptake, media penetration through micropores (osmosis) causing matrix swelling, and/or the degradation/erosion of the PLGA polymer [37]. The PLGA matrix properties that drive these mechanisms are the drug type, polymer MW, L/G monomer ratio, terminal end-cap type, and blockiness. All of these affect the delivery system’s physiochemical properties, degradation rate of the PLGA matrix, subsequent desired dosage, and drug release rate. While these properties provide considerable tunability for various drug delivery systems with drug release rates from days or weeks to a few months, the inherent heterogeneity of PLGA polymers makes the quality control aspects of the delivery systems a challenge [38,39]. With these properties in mind, two Lactel^®^ PLGA polymers characterized with inherent viscosities in the range of 0.15–0.25 dL/g, a lactide–glycolide (L/G) monomer ratio at 50:50 and end-groups with either acid or ester termination, and similar in nature to the Ozurdex PLGA polymers were selected for the studies herein. Further, it was theorized that with these two polymers, three 20% *w*/*w* dexamethasone-loaded formulations and three without dexamethasone formulations could be prepared to deliver fast (100% acid end-capped), intermediate (80%/20% acid–ester end-capped), and slow (60%/40% acid–ester end-capped) theoretical drug release implants.

The manufacturing process for the implants was a single-pass hot-melt extrusion (HME) process. The implant diameters obtained were in the range of 0.375 to 0.420 mm. For characterization and the in vitro release studies, only HME implants in the range of 0.400 to 0.420 mm were selected. Implants in this diameter range fit into the 22-gauge needles that are typically used in ophthalmic medical clinics for intravitreal injections (IVT) [8]. With diameters in this range, the implants could be loaded and ejected from our lab’s custom-made needle-in-needle injector. Additionally, although an automatic implant cutter was purchased, it was not compatible with the HAAKE mini-CTW (HME instrument) used in this study, which was an instrument for lab-scale manufacturing, so implants were cut manually using a Personna Surgical Blade (American Safety Razor Company, Staunton, VA, USA). The HME instrument allowed for a maximum batch size of 10 g for each of the dexamethasone-loaded and polymer-only composition formulations. The combined batches produced ~115 strands (each ~6 to 8 inches in length) that closely met our criteria. Consequently, this manufacturing method provided a very high throughput for implant production. The die plate with an aperture size of 0.4 mm was not found to produce implants with 0.4 mm diameters. Instead, implants produced with this aperture size manifested larger diameters, possibly due to extrudate strand expansion during the cooling and solidification on the conveyor. Consequently, a die plate with an aperture of 0.3 mm was used to produce implants of the desired diameter even though toward the end of the process, implant diameters declined. Another study discussed the adjustments that were made to the conveyor belt speed and rpm of the extruder’s barrel screw to attain implants with more consistent diameters in the 0.400 to 0.420 mm range throughout the entire manufacturing process [7].

### 4.2. Physiochemical Characterizations

#### 4.2.1. Implant Dexamethasone Content Determined by HPLC

Implants appeared uniform as they were manually cut to 6 mm lengths. The actual dexamethasone content was determined using HPLC with manual integration; the values obtained were 19.71 ± 1.05, 19.56 ± 0.46, and 19.25 ± 0.92% (*w*/*w*) for the HME-1, HME-2, and HME-3 implants, respectively. These results are in good agreement with the theoretical target concentration of 20% dexamethasone (*w*/*w*) for each of the drug-loaded implant formulations.

#### 4.2.2. Thermal Analysis—Glass Transition Temperature (Tg) for Implants and Formulation Components Using Differential Scanning Calorimetry (DSC)

**Figure 1:**
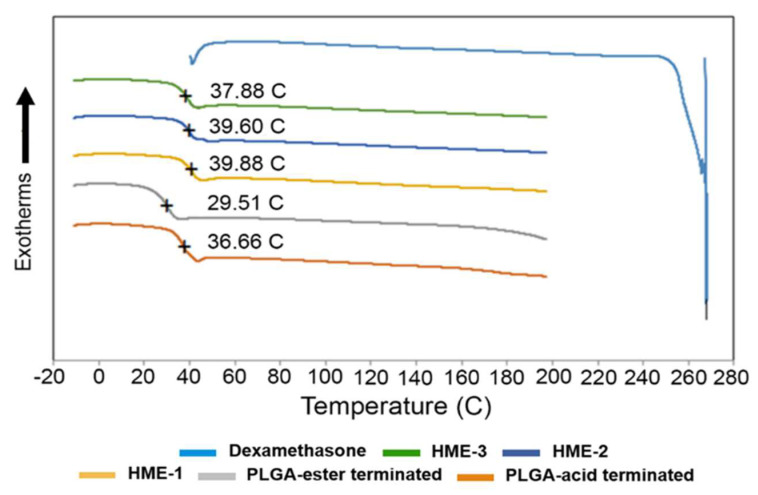
DSC thermograms of dexamethasone, implant dexamethasone formulations HME-1 to 3, PLGA acid, and ester end-capped formulation components.

To measure T_g_, a heating–cooling rate of 20 °C/min was applied to all samples during DSC analysis. By applying the first heating step in the process, the thermal/heat history of the samples was erased. The melting point of dexamethasone, T_g_ of PLGAs, and dexamethasone implants are marked in the DSC thermograms (Figure 1). The melting point of dexamethasone was determined to be 267.83 °C. However, the dexamethasone endothermic peak was only observed during the first heating cycle. This can be explained by the dexamethasone degrading after reaching its melting temperature (T_m_). Once the drug started to degrade around 270 °C, the structural changes were irreversible, which is evidenced by the lack of its recrystallization. Therefore, a second heating cycle was used for all the other components except for the drug dexamethasone. Also, the polymeric compositions exhibited a gradual deviation from baseline in the 30 to 40 °C temperature range, with the midpoint representing the T_g_ of the material.

It has been reported elsewhere that the glass transition temperatures (T_g_) of PLGA polymers are above the physiological temperature of 37 °C, because of their rigid chain structure in a glassy state [39]. Based on our DSC analysis, the Tg of the two PLGA polymers measured indicated that there was only around a 7 °C difference between them. The lowest value was recorded for the ester end-capped polymer, and both polymers had a Tg slightly lower than physiological temperature (Figure 1). Also, increasing the percentage of the ester end-capped PLGA polymer lowered the T_g_ values of the drug-loaded formulations. As for the T_g_ of the dexamethasone-loaded implant formulations, they can be arranged as follows: HME-1 >HME-2 >HME-3. A higher T_g_ is an indication of a less mobile polymer chain. When a direct comparison between the acid end-capped PLGA polymer and HME-1 implant was performed, the T_g_ values were 36.7 and 39.9 °C, respectively (Figure 1). It is possible that the dexamethasone particles could have restricted the rotational motion within the PLGA polymer chains and consequently increased the T_g_ value.

The effect of Tg on the crystallinity of the polymer composition has caused considerable debate in the past regarding its indirect effects on the biodegradation rate [39,40]. Some researchers have suggested that the polymer degrades faster with a decrease in crystallinity due to an increased rate of hydration and hydrolysis [39,41,42]. Regardless of all the factors involved, a reduction in Tg during release is consistent with a decrease in the polymer molecular weight due to degradation, which, in turn, causes the drug within the matrix to be released [38,43].

### 4.3. Physicomechanical Characterizations

#### 4.3.1. Melt Viscosity of Implant Compositions

The melt viscosity was used to set the HME manufacturing process parameters for speed and temperature. The melt viscosities of the implant compositions at three temperatures (e.g., 60 °C, 80 °C, and 100 °C), based on the mean torque values at a screw speed of 20 rpm, showed that the highest temperature (100 °C) provided the lowest torque values regardless of the implant formulation. The mean torque values were recorded by the hot-melt extruder instrument’s software. It collected 120 readings during the test period (Supplemental Figure S1).

A decrease in the mean torque is reflective of a reduction in the viscosity of the molten implant formulation compositions. When the temperature was increased from 60 °C to 80 °C, the most significant decrease in the mean torque values or viscosity was noted for all the formulations. Then, there was a further viscosity decrease but to a lesser degree once the temperature was elevated to the highest temperature (100 °C). Formulations loaded with dexamethasone (HME-1, HME-2, and HME-3) exhibited increased mean torque values or viscosity by all three formulations, with major differences observed when the temperatures were below 100 °C. Therefore, with an increase in temperature, the mean torque values needed for instrument operation decreased. A temperature setting of 100 °C as opposed to 60 °C was deemed suitable for processing the molten implant materials so that they were within the torque limits specified by the HME manufacturer (Supplemental Figures S1 and S2). Furthermore, since the melting point of dexamethasone is ~262–268 °C [44], at the processing temperature, dexamethasone was expected to remain in its crystalline structure, which proved to be a valid assumption due to the conformation provided by the PLM and XRD analysis. 

Additionally, the torque value of the dexamethasone-loaded HME-1 formulation was about twofold that of its counterpart, HME-4, the drug-free version of the HME-1 formulation, at the lowest temperature setting of 60 °C. During the attempt to process it at 60 °C, the barrel screws stopped rotating due to the high viscosity, and the test was terminated after only 28 recordings per second were recorded. Even though 60 °C is above the Tg of the PLGA polymer, it was not sufficient to create a usable molten material for the drug-loaded HME-1 formulation. Meanwhile, the HME-2 and HME-3 formulations had lower torque values at 60 °C, so all 120 readings were obtained for each formulation at the three temperature settings (e.g., 60°, 80°, and 100 °C). In other runs when the temperature was kept constant at 100 °C, and the screw speed was increased from 20 to 100 rpm, the torque values also increased (Supplemental Figure S2). Because of these observations, it was decided that the desired manufacturing parameters would be to set the screw speed at 20 rpm and screw barrel temperature at 100 °C for the single-pass HME manufacturing implant manufacturing process.

#### 4.3.2. Three-Point Bend (TPB) Results

##### Influence of Humidity on Moisture Adsorption and TPB Mechanical Properties

To obtain the moisture adsorption weights for the implant formulations stored under high-relative humidity conditions, representative implant samples were removed after 24, 48, 72, and 96 h from the humidifier, weighed, and the average percent moisture adsorption calculated (Supplemental Figure S3).

**Figure 2:**
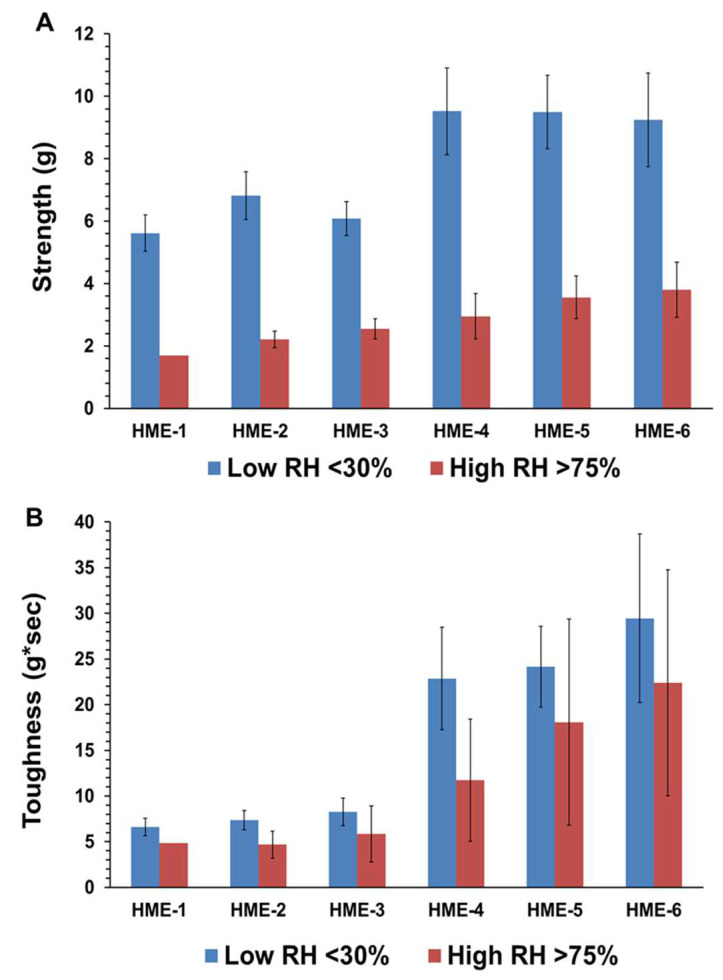
(**A**) is the average strength (g), and (**B)** is the average toughness (g*sec) for with/without dexamethasone implants stored at low or high relative humidity. Mean ± STDEV, *n* = 6.

As for the influence of humidity and TPB analysis for the HME-1, HME-2, and HME-3 implant groups and the drug-free implant groups (HME-4, HME-5, and HME-6), the drug-free implant groups exhibited greater breaking force as well as toughness relative to their dexamethasone-loaded counterparts. Implants stored at a high relative humidity of about 75% RH showed lower strength breaking forces when compared to those stored at low relative humidity. Implants stored in a desiccator exhibited greater mechanical strength as opposed to those kept at about 75% RH, (Figure 2A,B). Similar but less prominent trends were evident for implant toughness. With the inclusion of dexamethasone, there was a decrease in the breaking strength force as well as toughness for the HME-1, HME-2, and HME-3 formulations, but all exhibited similar values for both parameters under high- and low-relative humidity conditions. However, in general, it can be stated that drug-loaded implant mechanical properties prior to release have no correlation to the results from the dexamethasone implant release study data. The physicomechanical characterization of implants indicated that the diameters and mechanical properties are comparable among formulations.

### 4.4. Surface Morphological Properties

#### 4.4.1. Surface Roughness of Dexamethasone-Loaded Implants by AFM

Two parameters, Rq and Ra, characterizing surface roughness, were determined for each implant sample, using atomic force microscopy (AFM) with the NanoScope Analysis v1.50 software package. These two parameters, Rq and Ra, are typically defined as the root mean square (RMS) average of height deviations from the mean image plane and the arithmetic average for the absolute values of the surface height deviations from the mean plane, respectively [45,46,47]. They are the two most used roughness statistics obtained from this type of analysis.

**Figure 3:**
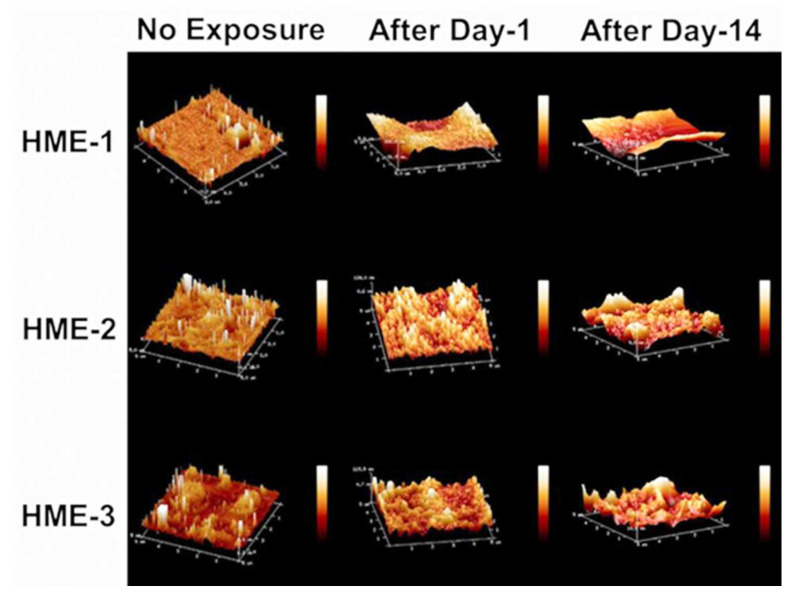
AFM images of dexamethasone implants from left to right, no exposure, after day-1, and after day-14 exposure to the PBS (pH 7.4) media.

**Figure 4:**
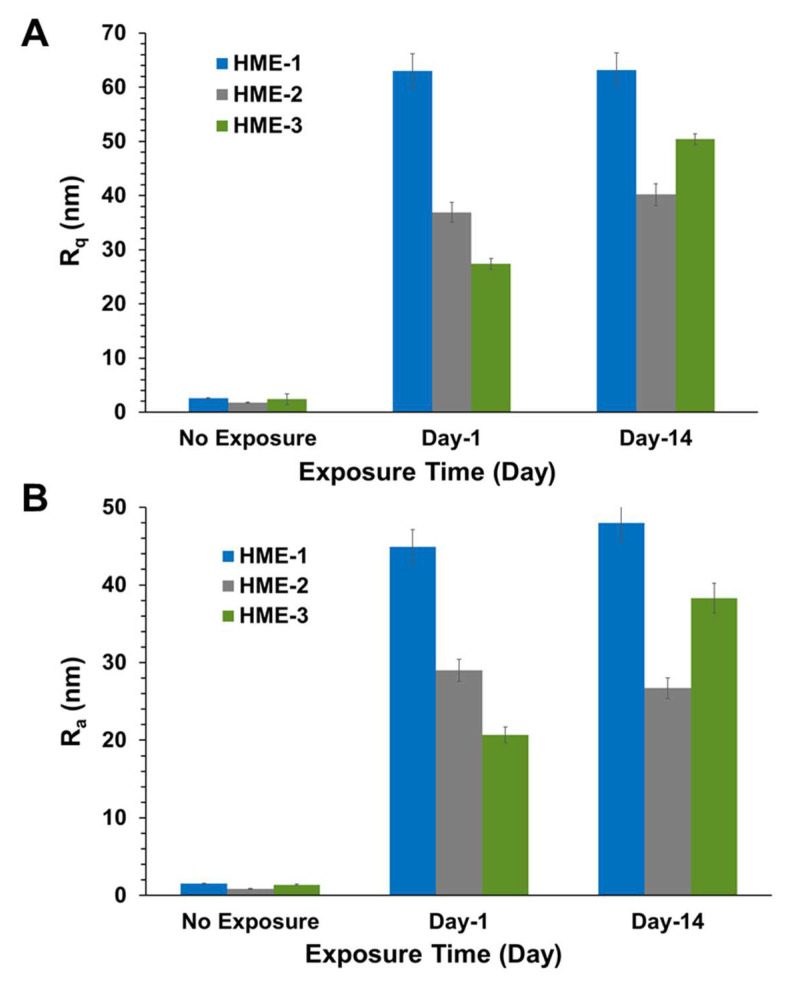
AFM surface roughness, (**A**) shows R_q_ and (**B**) shows R_a_ values for implants after exposure to PBS. Mean ± STDEV, *n* = 3.

Three 5 µm regions of each implant were imaged by AFM at random to obtain Rq, Ra value averages, and standard deviation values (*n* = 3) for these parameters. The 3D AFM images and the quantification of implant surface roughness by AFM are presented in Figure 3 and Figure 4A,B. Regardless of the formulation compositions, all dexamethasone implants had very smooth surfaces after manufacture and before exposure to drug release medium, PBS pH 7.4.

Intact implants exhibited Rq and Ra values in the range of 1 to 2.5 nm. After only day-1 exposure to PBS pH 7.4, these dimensions increased by more than 10-fold for all formulations, with the values being the highest for the HME-1 formulation at 63 and 45 nm for Rq and Ra, respectively. These values were greater than those of the HME-2 and HME-3 formulations (Figure 3).

While the surface deformations remained the highest for HME-1, the values for HME-2 and HME-3 increased between day-1 and day-14 with HME-3 increasing the most. Therefore, it appears that surface deformation is faster for the more hydrophilic composition and slower for the more hydrophobic compositions due to sterically hindered hydrolysis.

#### 4.4.2. Crystalline Structure of Implants with/without Dexamethasone Based on PLM and XRD

**Figure 5:**
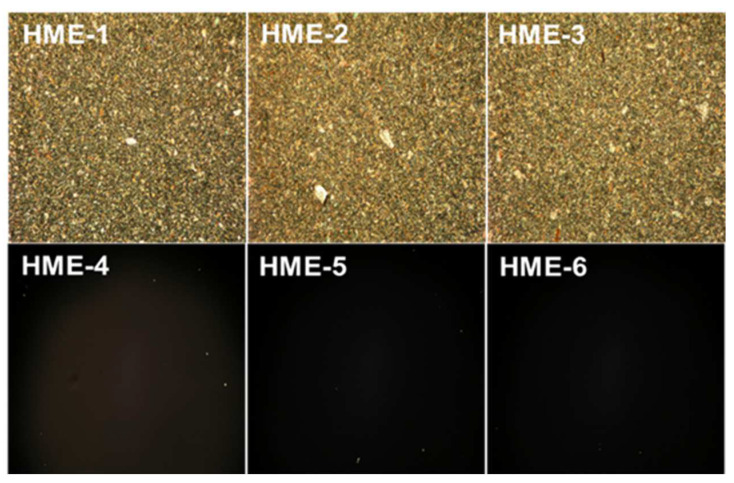
Polarized light microscopy (PLM) images of dexamethasone/without drug implant films using a melt-congealing technique at 10× magnification.

For the polarization light microscopy (PLM) studies, the melt-congealing sample preparation method was better than the solvent film casting method because the dexamethasone crystalline structure was unchanged after sample preparation using the melt-congealing approach. The images from polarized light microscopy (PLM) revealed that dexamethasone implant films (HME-1, HME-2, and HME-3) showed aesthetic birefringence in a two-phase system owing to the presence of drug crystals or crystallites evenly encapsulated throughout the film casts because of the thorough blending during the hot-melt extrusion process (Figure 5). Meanwhile, the three PLGA polymer film casts of HME-4, HME-5, and HME-6 without dexamethasone were not birefringent or crystalline in nature (evidenced by no crystals being visible in the polymer film), which resulted in no PLM signal (Figure 5) [48].

**Figure 6:**
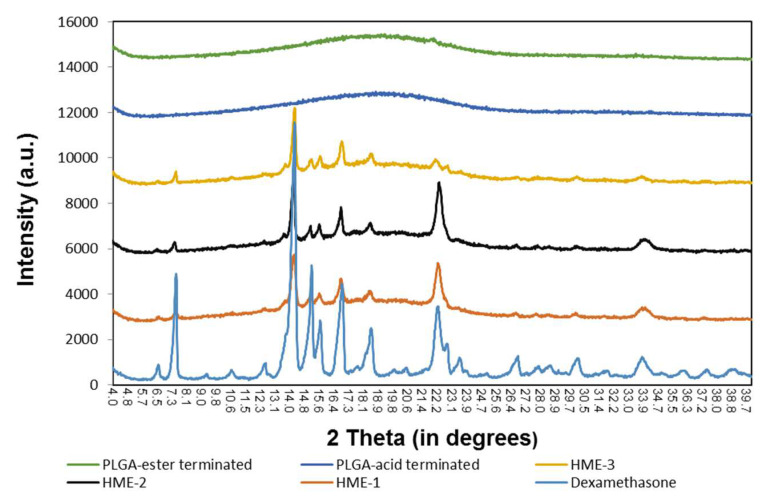
The X-ray diffraction patterns of the polymers, implant drug formulations, and the drug dexamethasone.

Similarly, the results of the X-ray diffraction (XRD) diffractograms (Figure 6) show that the PLGA polymers exhibited “halo” patterns without any visible diffraction peaks, while dexamethasone exhibited multiple dominating diffraction peaks at 2θ ranging from 4 to 40 degrees [24,49,50,51,52]. These dominating peaks were also identified in the dexamethasone implants (HME-1, HME-2, and HME-3) suggesting that crystals of dexamethasone were embedded uniformly in the amorphous PLGA matrices. When comparing the XRD patterns of pure dexamethasone and dexamethasone implants, the peak intensity was weaker in all the dexamethasone–polymer implant formulations. The diffractograms also indicated that dexamethasone was present in the crystalline state in all three implant formulations. In a detailed comparison between dexamethasone implants, XRD patterns showed strong similarities between HME-1 and HME-2. However, the intensities of two peaks (e.g., at 22.2° and 33.9°, especially the 22.2° peak) decreased in the HME-3 formulation when compared to the other thermal patterns. This may be due to the higher level of ester end-capped PLGA in the HME-3 formulation and/or a possible change in the orientation of drug crystals.

#### 4.4.3. Scanning Electron Microscopy (SEM) Imaging of HME Implants

**Figure 7:**
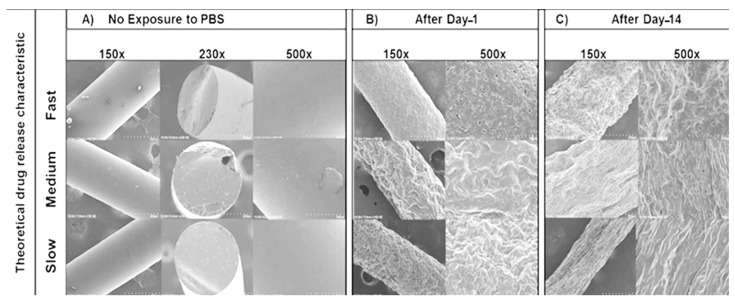
SEM images of samples under 150×, 230×, and 500× magnifications. (**A**) No exposure to PBS, (**B**) after Day-1, and (**C**) after Day-14.

The SEM images initially showed smooth surfaces for all implants after manufacturing (Figure 7A). After day-1 immersion in PBS (Figure 7B), there were more surface perforations on the HME-1 implant than HME-2 and HME-3. Conversely, the HME-2 and HME-3 implant formulations appeared to exhibit more surface degradation than pores at the same time points (Figure 7A–C). After day-14 (Figure 7C), the pores of HME-1 appeared to have sealed, while the surfaces of all implants were further degraded and/or eroded. However, all implant formulations showed similar surface degradation and pores.

The results observed from the atomic force microscopy (AFM) and scanning electron microscopy (SEM) imaging can to some degree be explained by the different end-cap functional groups of the two PLGA polymers selected for the studies herein. HME-1 only incorporated the acid-capped PLGA polymer into the formulation, which on exposure to the media initiated rapid swelling through micropores probably caused by the monomer sequence [37,53,54,55]. Some researchers have extensively studied polymer sequence heterogeneity and found that the increased number or lengths of glycolic acid segments leads to a significant increase in swelling, hydrolysis, and erosion [55]. Additionally, regarding HME-2 and HME-3, it is generally accepted that ester end-capped polymers can dramatically reduce degradation/erosion compared to acid end-capped polymers, which typically translate to a slower drug release [56,57]. Unfortunately, resin manufacturers can use different ester end-caps, which are not typically reported but have been known to further affect polymer hydrolysis, complicating the drug formulation development [37,58]. However, several recent studies have shown that other factors also come into play when trying to predict the mechanism for polymer degradation/erosion and drug release. Such factors include the drug type, polymer synthesis method (polycondensation vs. ring-opening polymerization, ROP), synthesis method processing parameters, arrangement of the LA/GA sequences of the lactide/lactic acid and glycolide/glycolic acid blocks commonly referred to as “blockiness” along the polymer chains, release media–temperature–pH, variable glass transition temperatures, and residual monomer percentage [37,38,59,60]. Even so, most still agree that the characterization of polymer properties should still focus on the polymer MW, L/G ratio, and terminal end-cap of the PLGA polymers [37,38,39,43].

### 4.5. In Vitro Dexamethasone Release Studies

#### 4.5.1. In Vitro Dexamethasone Release Testing

Implant release characteristics deviated from their theoretical predictions. A comparison of the in vitro release profiles for the three different dexamethasone–PLGA implant formulations (HME-1, HME-2, and HME-3), with three different batches of each formulation, and quadruple samples taken from each batch was performed. A burst release of dexamethasone was observed at early time points for the HME-1 formulation, while the HME-2 and HME-3 implants had no apparent burst release and an overall lower rate of release. The HME-1 release profile appeared to follow the more typical triphasic PLGA implant formulation profile (initial burst release, lag phase, and then followed by a zero-order release phase) than the other two formulations. The initial burst release for drug delivery devices is an important aspect of the drug release process, as it is related not only to the polymer matrix composition but also to the drug properties, drug concentration, and polymer hydrophobicity [39]. Drug delivery matrices with lower polymer–drug ratios tend to exhibit larger initial burst releases compared to matrices with higher polymer–drug ratios [39]. If this initial burst is very large, it could be potentially toxic and should always be evaluated for each drug delivery system [39].

**Figure 8:**
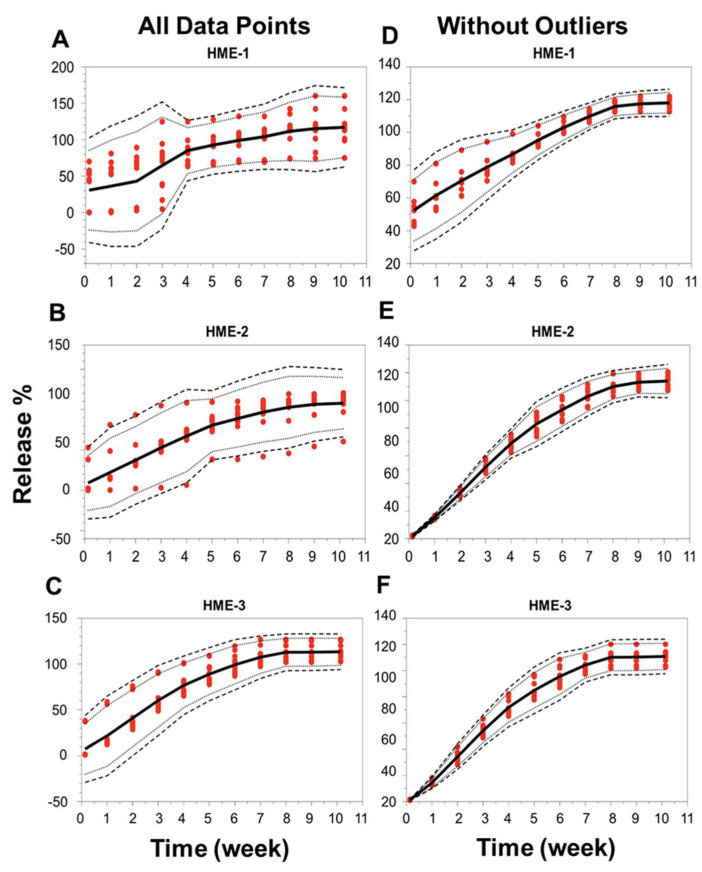
Release profile similarities. From three different batches manufactured using three different formulation compositions of the drug dexamethasone–PLGA implants analyzing quadruple samples (**A**–**F**), each formulation with all data points (panels on the left side or without outliers’ panels on the right side). The 99% TLs (black dashed line, *n* = 12) were defined with 95% confidence (black dotted line, *n* = 12). Release values from the test samples (red circles, *n* = 12). The mean is the solid black line. TL, tolerance limit.

When the focus was placed on the analysis of individual implants, the release profiles deviated under the conditions of our study for 5 out of 12 implants in the HME-1 group (samples B1-2 and 4, B2-2 and 4, and B3-3), 3 out of 12 in the HME-2 group (samples B1-4 and B2-1 and 3), and 2 out of 12 in the HME-3 group (samples B2-1 and B3-1). These implants that deviated from the norm were observed to have stuck to the wall of the release container at or below the PBS release media level for at least a portion of the incubation period. This is probably not due to manufacturing differences but more likely due to the release study method (e.g., the agitation caused by the incubator), which may have forced the collision of these implants with the walls of the container after the polymer started to degrade/erode, providing a soft tacky surface that resulted in the adhesion of the implant to the wall. These encounters with the container walls could have caused implant morphological changes, which may have affected the polymer degradation and drug release kinetics. The localized pH of the release media around or within the implant could have varied, causing increased drug release or the lack thereof [61]. However, it is unclear if the release of dexamethasone was influenced by the implants being stuck to the container or by a manufacturing process-related deviation. These implants were considered outliers due to variance in the release compared to implants that remained in the solution. For implants in the HME-1 group, the outliers primarily exhibited a delayed release, HME-2 outliers showed no consistent release trend, and the HME-3 group’s outliers exhibited a faster release profile. The total percent (%) dexamethasone in the solution is shown in Supplemental Figure S4. The average cumulative dexamethasone levels for the in vitro release medium reached peak levels by day-21, -28, and -35 for the HME-1, HME-2, and HME-3 groups, respectively (Supplemental Figure S5). These levels then started to show a definite decline by day-42 with respect to each formulation and the lowest levels for the HME-1 group by day-71. The cumulative drug release data corrected for drug degradation (using 0.04 day-1 for the degradation rate constant from Matter and Kompella, 2019) showed a steady increase in dexamethasone release regardless of batch for the HME-2 and HME-3 groups with more variability for the HME-1 group (Supplemental Figure S6). When considering the day-71 averages corrected for dexamethasone degradation, the results were 117%, 113%, and 107% for the HME-1, HME-3, and HME-2 groups, respectively (Supplemental Figure S7). Additionally, a comparison of the in vitro release profiles for the three different dexamethasone–PLGA implant formulations (HME-1, HME-2, and HME-3), three different batches of each, and quadruple samples taken from each batch was performed using the model-independent tolerance limit method described by Martinez and Zhao (Figure 8A–F) [62]. The left side of Figure 8A–C shows all data points, whereas the right side is without the outliers listed previously, Figure 8D–F. Since the data points from the release profiles are within the tolerance limit ± 15% described by Martinez and Zhao for each composition (with and without outliers), it can be concluded that the different batches behaved similarly for each composition [62].

#### 4.5.2. Correlation of Implant Physicomechanical Properties with Dexamethasone Release

**Figure 9:**
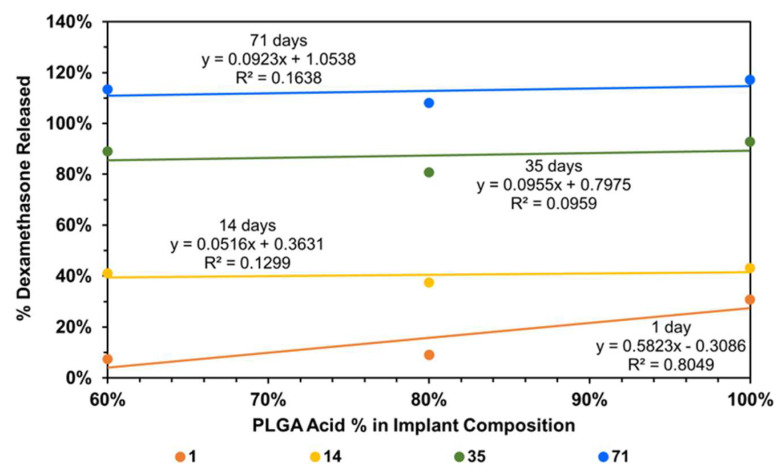
Correlation of cumulative dexamethasone release at end of day-1, -14, -35, and -71 with implant composition.

To understand the factors influencing drug release from implants, implant composition was correlated with dexamethasone drug release, using all data without any outlier omission (Figure 9). It is evident that the day-1 dexamethasone release generally increases with an increase in the content of the PLGA acid end-capped copolymer in the implant relative to the PLGA ester end-capped copolymer, with the correlation being high for day-1 dexamethasone release (R^2^ = 0.805) and low on other days (R^2^ < 0.164). Without outliers, the correlations improved dramatically for other days (R^2^ = 0.619 to 0.995) and decreased slightly for day-1 (R^2^ = 0.760; plots not shown).

**Figure 10:**
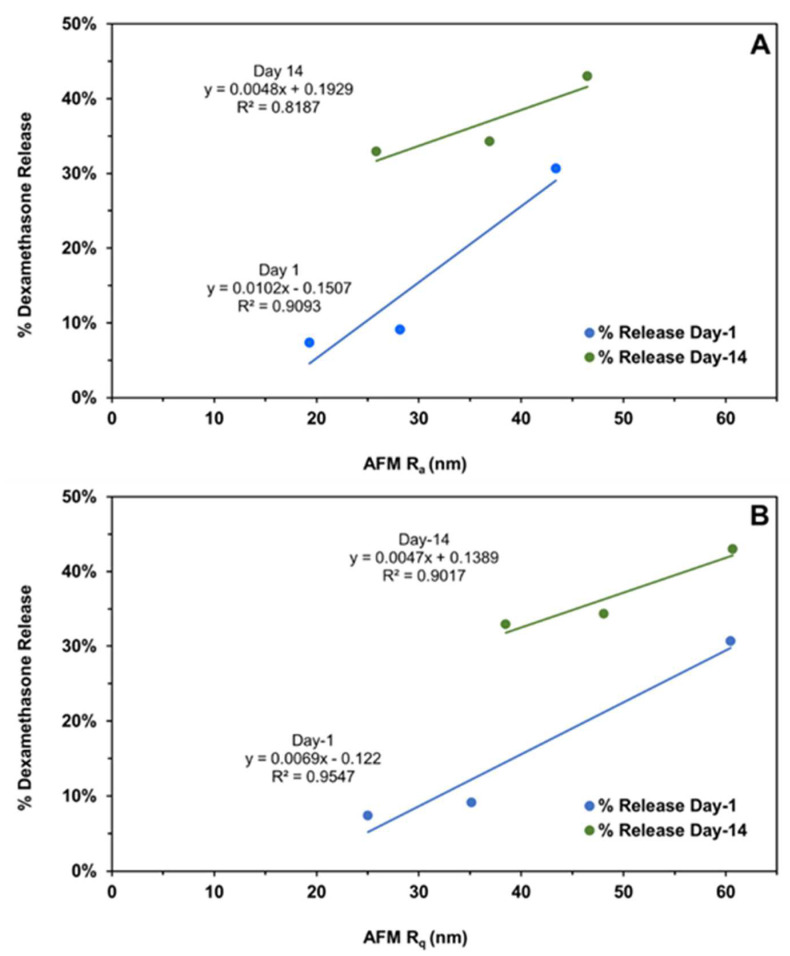
The correlation of the cumulative dexamethasone release at the end of day-1 and day-14 with the (**A**) implant average surface roughness (R_a_) and (**B**) root mean square roughness (R_q_) measured on the same days using atomic force microscopy. Different roughness measures represent distinct implant compositions, as shown in Figure 4.

**Figure 11:**
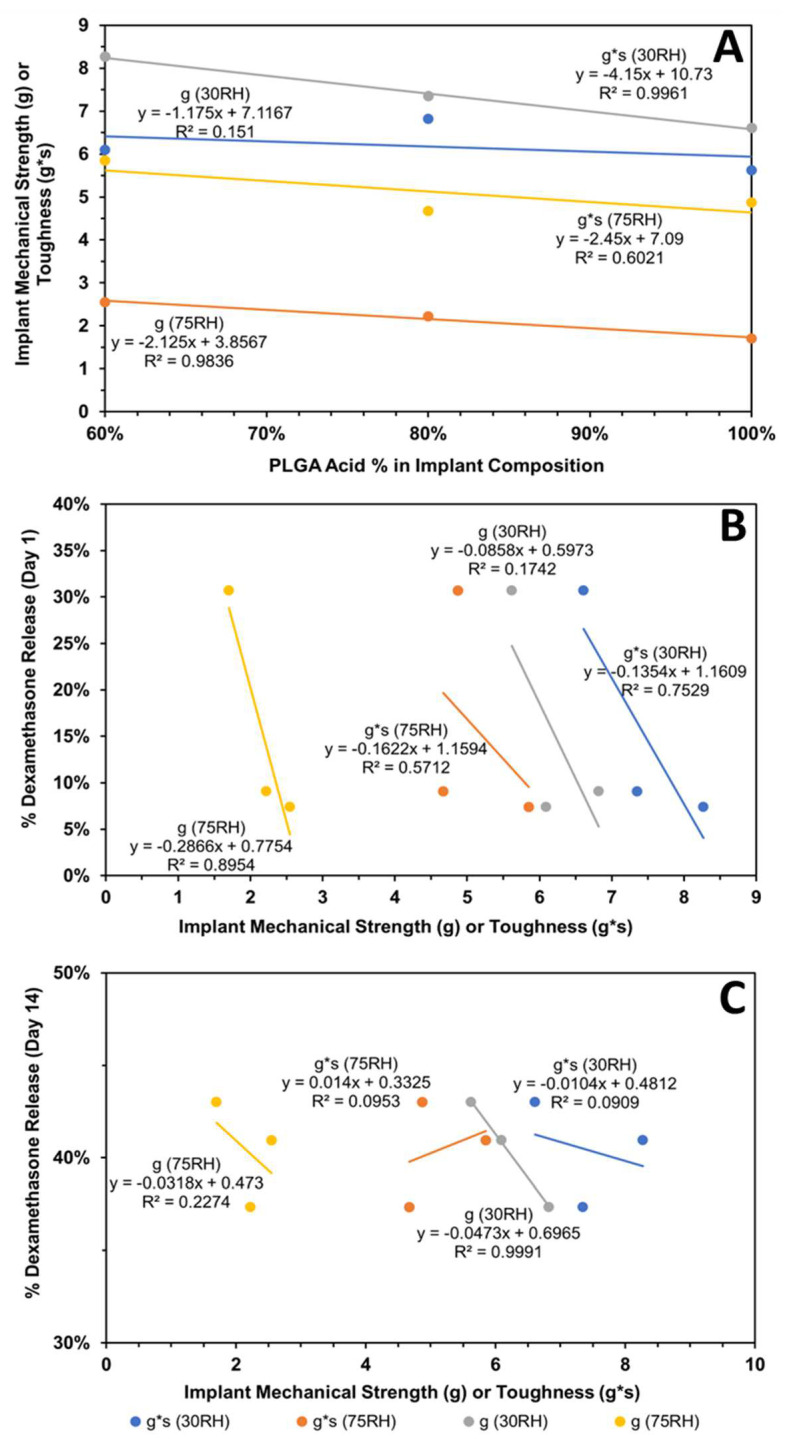
Influence of implant composition and relative humidity (RH) on its mechanical properties and dexamethasone release. Correlation of (**A**) implant mechanical properties (strength and toughness) with implant composition, (**B**) cumulative dexamethasone release at end of day-1 with mechanical properties, and (**C**) cumulative dexamethasone release at end of day-14 with mechanical properties. Mechanical properties were measured after storage at relative humidity of about 30 or 75%.

The subsequent correlations with mechanical properties are reported for all data without outlier omission (Figure 10A,B and Figure 11A–C). It is interesting to note that implant roughness (Ra as well as Rq) correlates with dexamethasone release on day-1 and -14, with a high coefficient of determination ranging from 0.819 to 0.955. The roughness measures correspond to implants incubated in the release medium for the same length of time. The correlation of implant mechanical properties, strength/toughness, with implant composition, relative humidity, and dexamethasone release is shown in Figure 11. With an increase in PLG acid content relative to PLG ester content, both the strength and toughness of implants tended to decline with good correlations in three (R2 ranged from 0.602 to 0.996) out of four cases (Figure 11A). With an increase in mechanical properties, dexamethasone release generally declined on day-1 (Figure 11B) as well as day-14 (Figure 11C), with the magnitude of the coefficient of determination as well as the slope being higher for day-1 correlations compared to day-14. That is, by day-14, the differences in the extent of release for implants based on mechanical properties were much reduced. The coefficient of determination for day-1 ranged from 0.571 to 0.895 in three cases (Figure 11B), and it was less than 0.227 in three cases and 0.999 in one case on day-14 (Figure 11C). In general, both strength and toughness exhibited similar trends in the above correlations.

## 5. Conclusions

This study demonstrated, using in vitro release tests (IVRTs), that biodegradable ophthalmic dexamethasone-loaded implants with 20% drug and 80% PLGA polymer(s) prepared using single-pass HME differ in physicochemical and/or physicomechanical properties and drug release depending on their PLGA polymer composition. An acid end-capped PLGA was mixed with an ester end-capped PLGA to make three formulations: HME-1, HME-2, and HME-3, containing 100%, 80%, and 60% w/w of the acid end-capped PLGA. In vitro release tests (IVRTs) indicated that HME-1 implants can be readily distinguished by their release profiles from HME-2 and HME-3, with the release being similar for HME-2 and HME-3. The implant release profiles for different implant compositions were similar between batches. On day-1 and day-14, the cumulative dexamethasone release correlated well with implant roughness parameters measured using AFM. Further, the composition of the implant correlated well with day-1 dexamethasone release, with the release increasing with an increase in PLGA acid content relative to PLGA ester content. The mechanical strength and toughness of implants decreased with an increase in the PLGA acid polymer content, and there was generally a good correlation between dexamethasone release and mechanical properties on day-1, with the differences and correlation diminishing on day-14.

IVRT designs as well as implant manufacturing processes contribute to differences in dexamethasone release. An IVRT study design should discriminate release profiles but should not contribute to any observed differences. Thus, the optimization of IVRTs is very important as it can contribute to the data variability and reliability of conclusions. Based on our release study, additional considerations should be placed if there is the adherence of implants to any surfaces during in vitro release and potentially even in vivo, as it may alter the drug’s release characteristics. The performance of in vivo implants under various settings (e.g., implant injection into different eye locations or when/if the implant is attached to lens or retinal surfaces or if the implant is deformed) should also be considered in these evaluations.

Despite all the research studies performed involving PLGA long-acting release (LAR) drug delivery systems which have showcased their many desirable properties and produced several products approved by the FDA, there remain areas of research that should be pursued to improve the viability of these systems. These areas of research might be best to focus on establishing therapeutic guidelines based on the drug type, manufacturing procedures/delivery method for specific devices, manufacturing procedures and properties of the polymers, and release media type/temperature. This is evidenced by the fact that at the present time, there are 19 PLGA-based LAR products approved by the U.S. Food and Drug Administration (FDA) that achieve controlled drug release from days or weeks up to months. Regrettably, despite their use and all the research with these systems, there are no approved generic drugs for these products. The primary causes for this phenomenon can be traced back to formulation complexities, minor differences in polymer properties (between venders and lots), and the unknown influences of manufacturing variables on product performance and the lack of process performance quality measures. Also, using in silico modeling in the design of experiments could have tremendous implications for faster product development and product introduction but also assist with the standardizing of manufacturing guidelines for the desired therapeutic products.

## Figures and Tables

**Table 1:** Implant formulations manufactured by hot-melt extrusion (HME) with/without the drug dexamethasone.

Formulations	Theoretical Release Characteristic	Dexamethasone (g)	PLGA–Acid End-Capped (g)	PLGA–Ester End-Capped (g)
HME-1	Fast	2	8.0	0
HME-2	Intermediate	2	6.4	1.6
HME-3	Slow	2	4.8	3.2
HME-4	Fast	0	10.0	0
HME-5	Intermediate	0	8.0	2.0
HME-6	Slow	0	6.0	4.0

**Table 2:** Three-point bend (TPB) test parameters.

Setting Description	Setting
Test Mode	Compression
Test Speed	0.1 mm/s
Target Mode	Distance
Gap Space	1.00 cm (0.394 in)
Stop Plot at	Target distance
Distance	5 mm

## Data Availability

The data presented in this study are available within the article or in the Supplementary Material.

## Supplementary Materials

The following supporting information can be downloaded at https://www.mdpi.com/article/10.3390/pharmaceutics16070895/s1, Figure S1: Melt viscosity of PLGA implants with dexamethasone (HME-1 to -3) and without dexamethasone (HME-4 to -6) at a fixed screw speed of 20 rpm and at three different temperature settings. Mean ± STDEV, *n* = 120 readings; Figure S2: Melt viscosity of dexamethasone–PLGA implant formulations at 100 °C and at three different screw speeds (RPM). Mean ± STDEV, *n* = 120 readings; Figure S3: Average moisture adsorption of PLGA implants with (HME-1 to -3) or without (HME-4 to -6) dexamethasone stored at high RH > 75%. Mean ± STDEV, *n* = 6; Figure S4: Percent (%) dexamethasone in solution from PLGA implants without correcting for dexamethasone degradation; Figure S5: Cumulative dexamethasone release of individual PLGA implants without correcting for dexamethasone degradation; Figure S6: Cumulative dexamethasone release from PLGA implants after correcting for dexamethasone degradation; Figure S7: Average cumulative drug release for three batches.
