# Supplementary material for: Hot-Melt Extrusion-Based Dexamethasone–PLGA Implants: Physicochemical, Physicomechanical, and Surface Morphological Properties and In Vitro Release Corrected for Drug Degradation
Source: Pharmaceutics. 2024 Jul 4;16(7):895. doi: 10.3390/pharmaceutics16070895 (PMC11280434; doi:10.3390/pharmaceutics16070895)
Supplement: Supplementary file 1 [file pharmaceutics-16-00895-s001.zip › pharmaceutics-3003100-supplementary.pdf]

Supplemental Figures

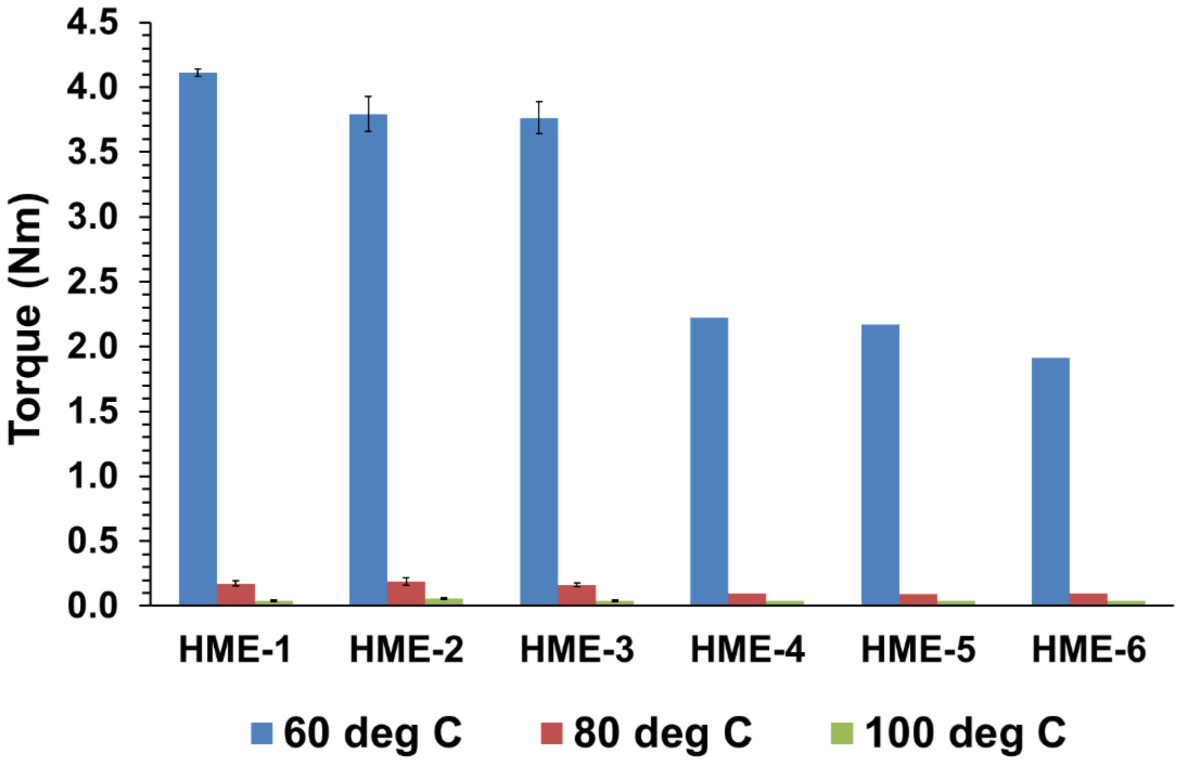

Supplemental Figure S1. Melt viscosity of PLGA implants with dexamethasone (HME-1 to 3) and without dexamethasone (HME-4 to 6) at a fixed screw speed of 20 rpm and at three different temperature settings. Mean ± STDEV, n=120 readings.

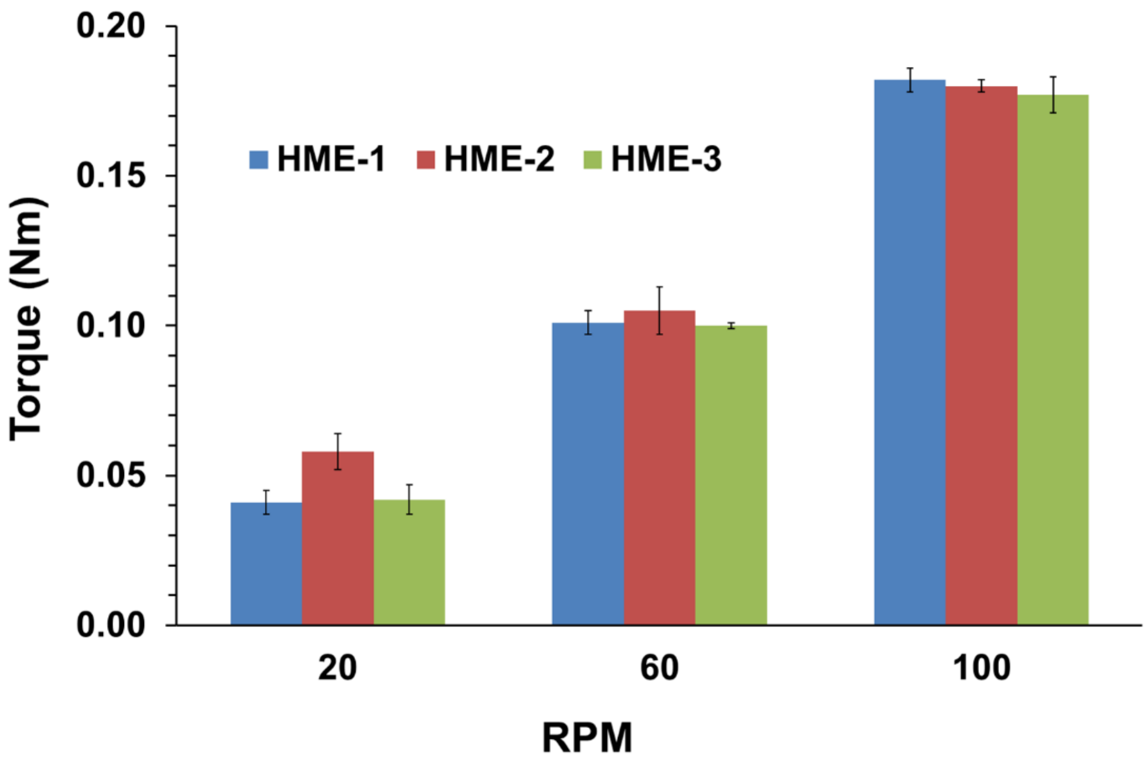

Supplemental Figure S2. Melt viscosity of dexamethasone-PLGA implant formulations at 100°C and at three different screw speeds (RPM). Mean ± STDEV, n=120 readings.

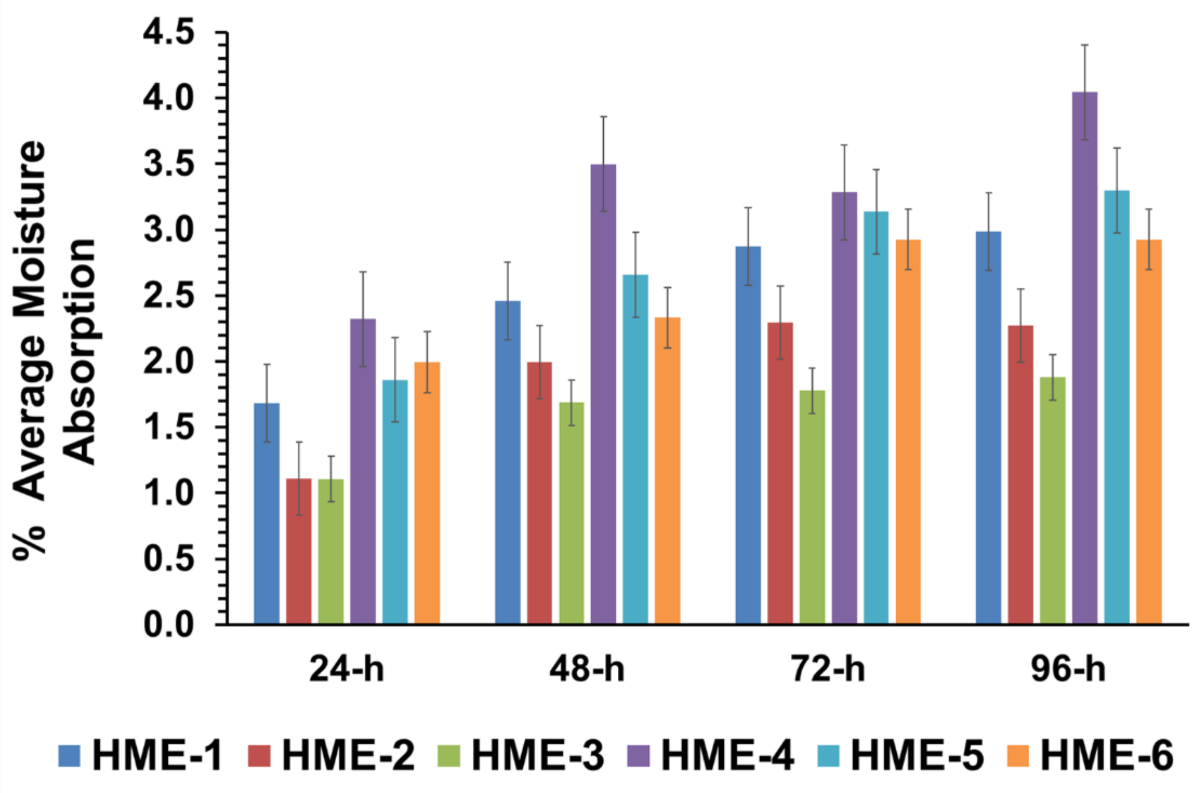

Supplemental Figure S3. Average moisture adsorption of PLGA implants with (HME-1 to 3) or without (HME-4 to 6) dexamethasone stored at high RH >75%. Mean ± STDEV, n=6.

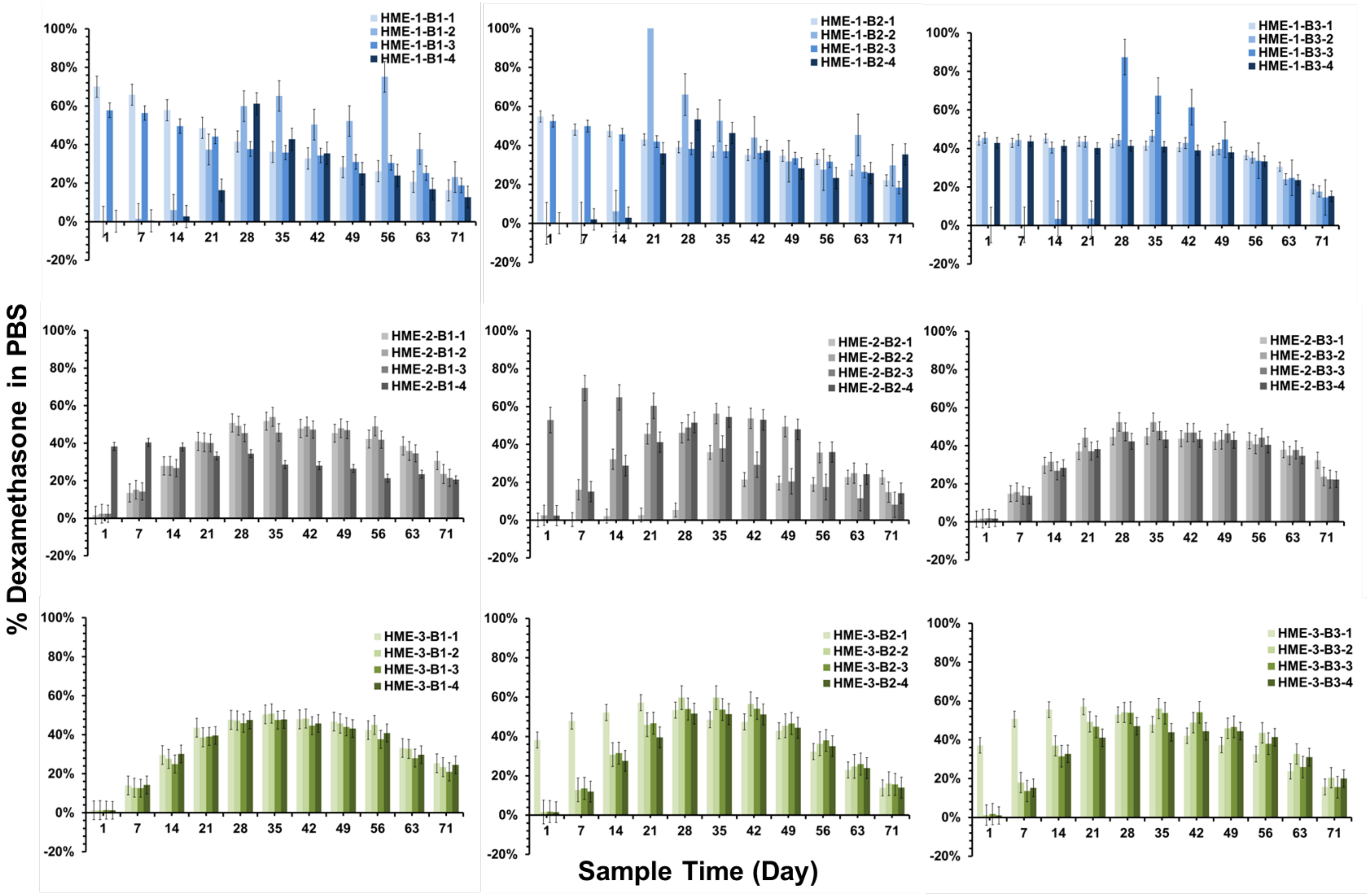

Supplemental Figure S4. Percent (%) dexamethasone in solution from PLGA implants without correcting for dexamethasone degradation. Percent drug release is shown from four different batches of HME-1, HME-2, and HME-3. The study was performed at 37°C in 100 mL PBS. Mean ± STDEV, n=4.

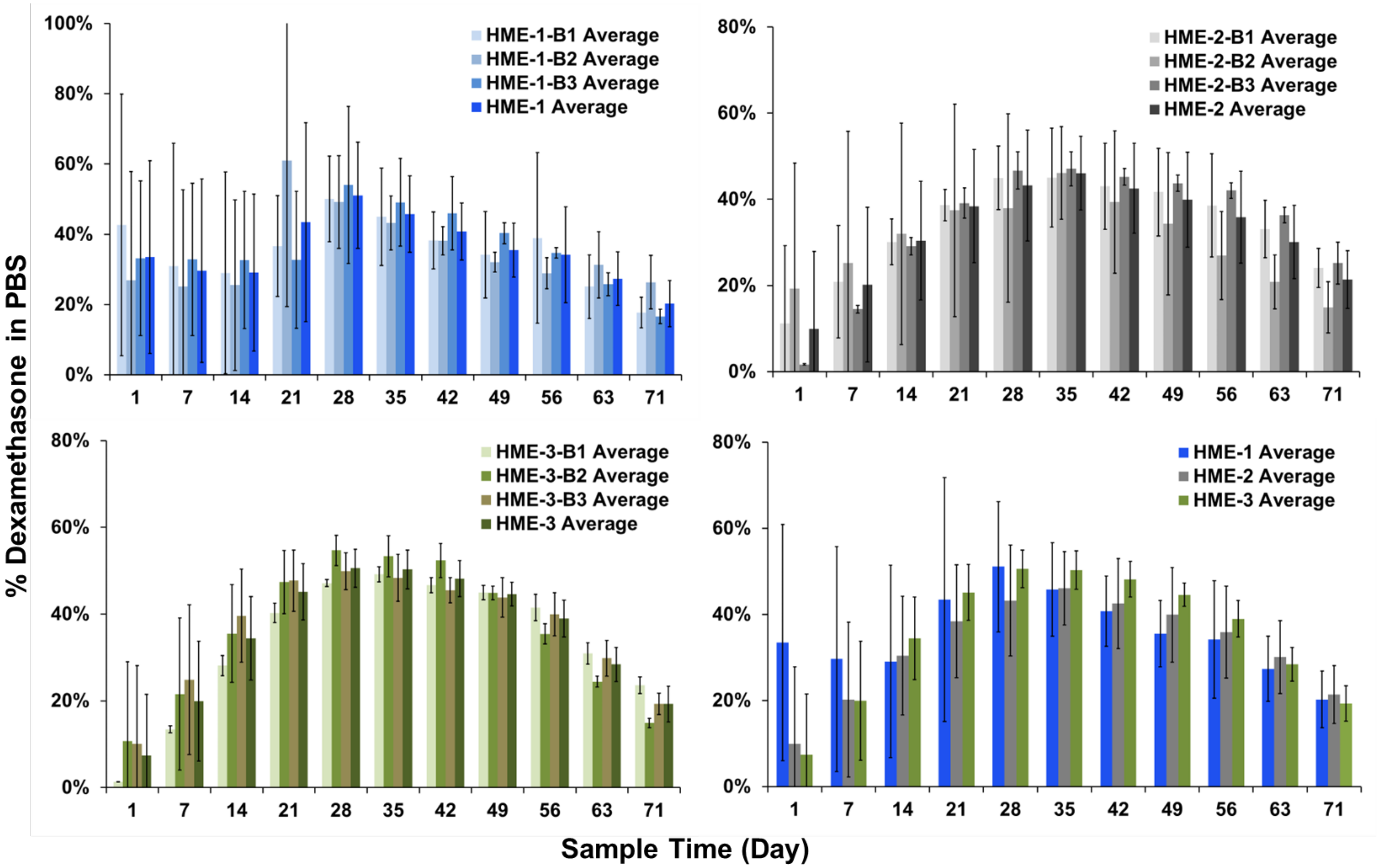

Supplemental Figure S5. Cumulative dexamethasone release of individual PLGA implants without correcting for dexamethasone degradation. Cumulative drug release is shown for three different batches of HME-1, HME-2, and HME-3. The study was performed at 37°C in 100 mL PBS. Mean ± STDEV, n=3.

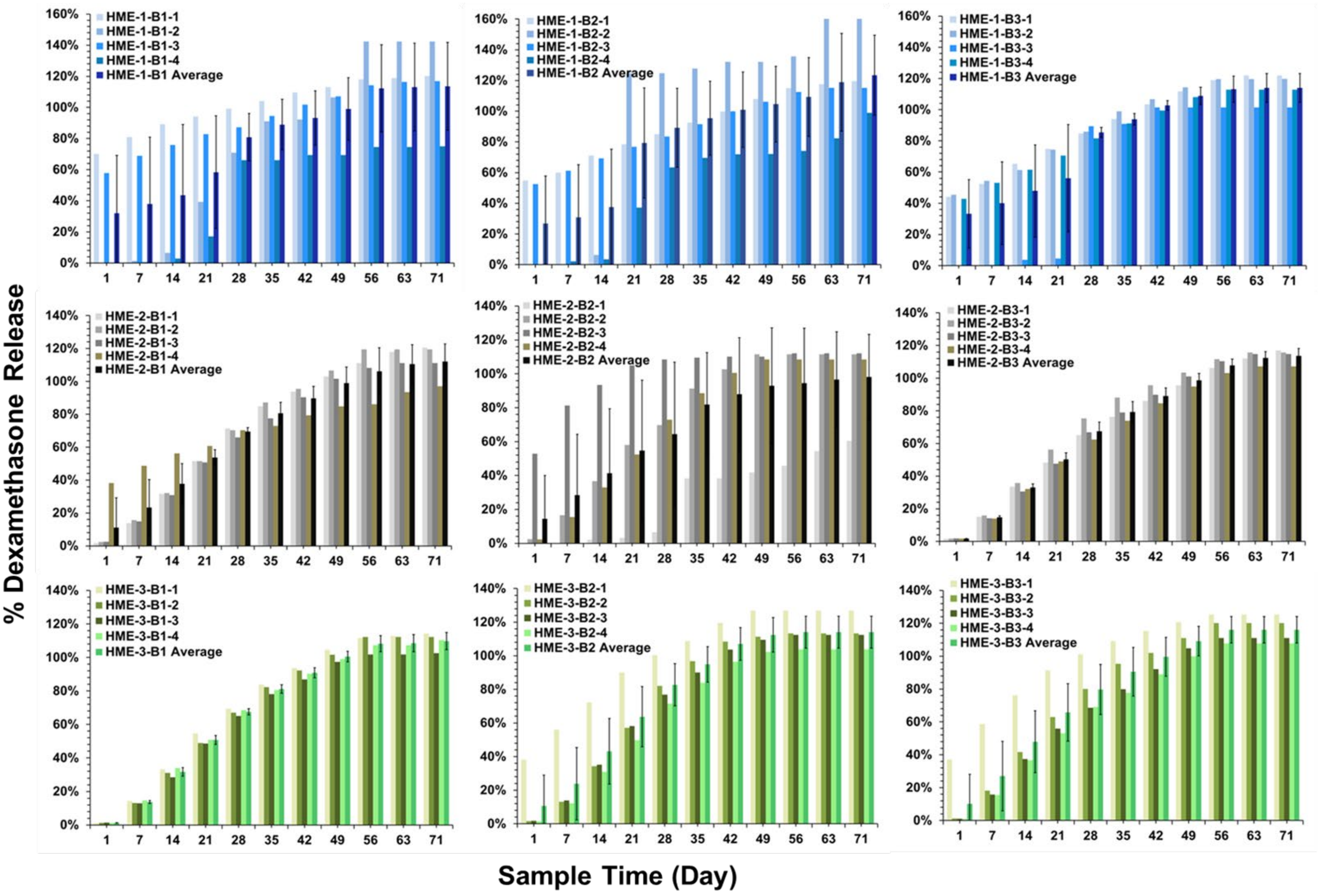

Supplemental Figure S6. Cumulative dexamethasone release from PLGA implants after correcting for dexamethasone degradation. The cumulative drug release of three different batches including release from individual implants. The study was performed at 37°C in 100 mL PBS. Mean ± STDEV, n=4.

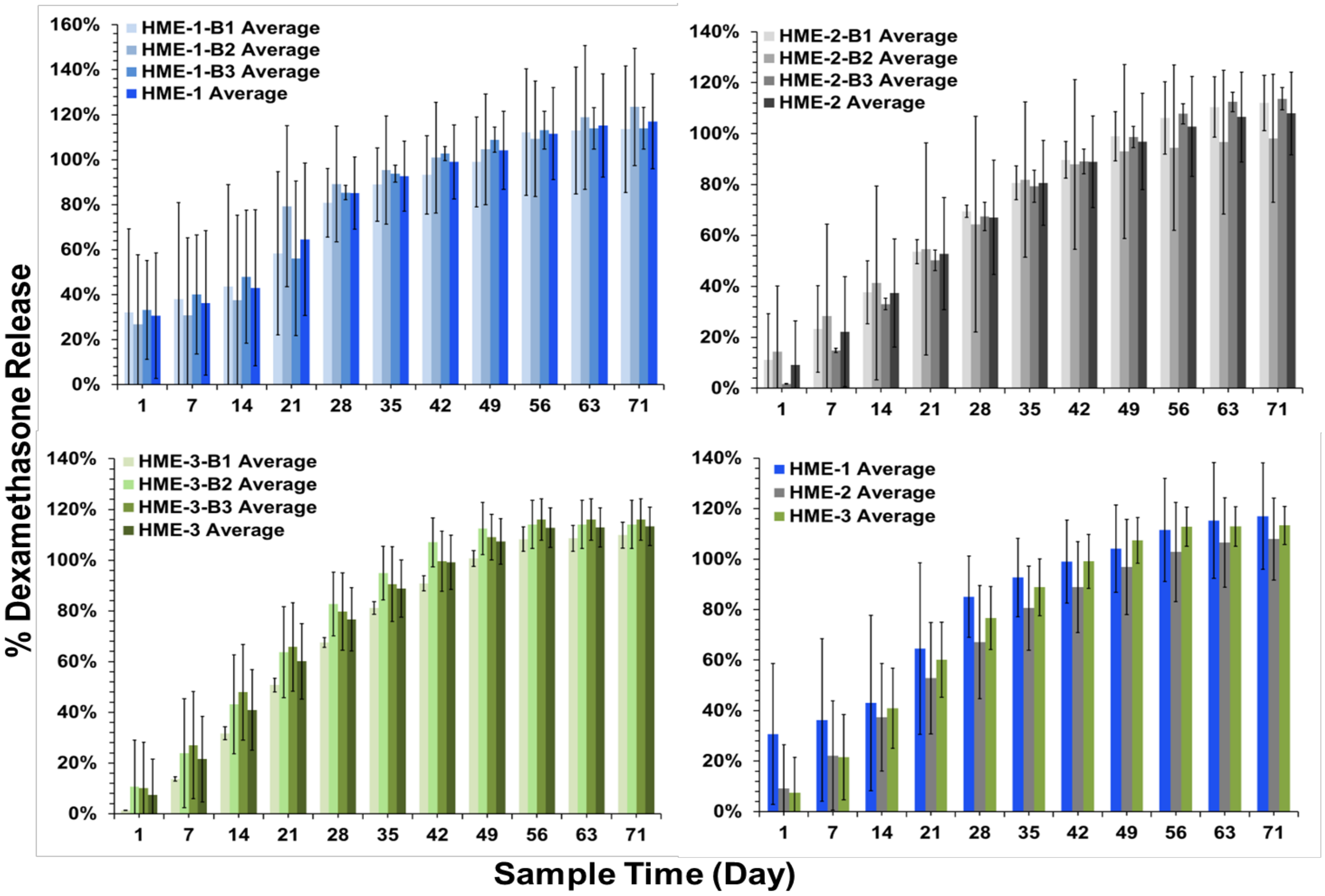

Supplemental Figure S7. Average cumulative drug release for the three batches. Release was corrected for dexamethasone degradation. The study was performed at 37°C in 100 mL PBS. Mean ± STDEV, n=3.
